# Ex vivo expansion of dysfunctional regulatory T lymphocytes restores suppressive function in Parkinson’s disease

**DOI:** 10.1038/s41531-021-00188-5

**Published:** 2021-05-13

**Authors:** Aaron D. Thome, Farah Atassi, Jinghong Wang, Alireza Faridar, Weihua Zhao, Jason R. Thonhoff, David R. Beers, Eugene C. Lai, Stanley H. Appel

**Affiliations:** Department of Neurology, Houston Methodist Neurological Institute, Houston Methodist Research Institute, Houston Methodist Hospital, Houston, TX USA

**Keywords:** Inflammation, Parkinson's disease, Immunotherapy, Neuroimmunology

## Abstract

Inflammation is a pathological hallmark of Parkinson’s disease (PD). Chronic pro-inflammatory responses contribute to the loss of neurons in the neurodegenerative process. The present study was undertaken to define the peripheral innate and adaptive immune contributions to inflammation in patients with PD. Immunophenotyping revealed a shift of peripheral myeloid and lymphoid cells towards a pro-inflammatory phenotype. Regulatory T cells (Tregs) were reduced in number, and their suppression of T responder proliferation decreased. The PD Tregs did not suppress activated pro-inflammatory myeloid cells. Ex vivo expansion of Tregs from patients with PD restored and enhanced their suppressive functions while expanded Tregs displayed increased expression of *foxp3*, *il2ra* (CD25), *nt5e* (CD73), *il10*, *il13*, *ctla4, pdcd1* (PD1), and *gzmb*. Collectively, these findings documented a shift towards a pro-inflammatory peripheral immune response in patients with PD; the loss of Treg suppressive functions may contribute significantly to this response, supporting PD as a disorder with extensive systemic pro-inflammatory responses. The restoration and enhancement of Treg suppressive functions following ex vivo expansion may provide a potential cell therapeutic approach for patients with PD.

## Introduction

Parkinson’s disease (PD) is a progressive, age-associated neurodegenerative disease characterized by the loss of dopaminergic neurons in the substantia nigra pars compacta (SNpc). The presence of alpha-synuclein (a-syn) inclusions, pathology in the basal ganglia, and multiple adversely affected systems throughout the body lead to a multitude of physical, sensory, and cognitive complications.^1,2^ Current studies and clinical trials focus on clearance of a-syn protein aggregates and disrupting cell–cell pathogenic protein transmission dynamics as hopeful avenues for therapeutic efficacy; however, successful results have been limited.^3–5^ Pathological findings and disease associations suggest that the immune system is intimately involved with PD, and an extensive study of the dysfunctional immune cells and their destructive mechanisms is warranted to generate novel, impactful therapeutic options with disease-modifying capabilities.

Clinical evidence points to inflammation and the immune response as an important mediator in PD pathogenesis and progression.^6–9^ Increased microgliosis and peripheral immune cell infiltration are noted in post mortem brain and in neuroimaging of patients with PD.^10–13^ Furthermore, pro-inflammatory cytokines (IL-6, TNF, IL-1β, IFN-γ etc.) are increased in the CNS as well as in the periphery.^14–19^ The genetics also point to the injurious effects of inflammation in PD as polymorphisms in a gene coding for HLA-DR (MHC class II surface receptor) increase the risk of late onset PD.^20^ Furthermore, other mutant genes linked to PD provide additional compelling evidence for the involvement of inflammation in disease pathogenesis such as PINK1, Parkin, and LRRK2.^21–25^ Epidemiologically, there is a decrease in risk of PD in people regularly using non-steroidal anti-inflammatories and in patients previously treated with anti-TNF therapies.^26–28^ Animal models of PD recapitulate these inflammatory findings and immune-modulating therapies are effective in ameliorating neuroinflammation, disease pathology, and subsequent neurodegeneration in these models.^29–34^

Due to these inflammatory associations in PD, peripheral immune cell dysfunction is a topic of interest and investigation over the years while also positioning PD as a prime candidate that would benefit from immune-modulating therapies.^35–37^ With regard to the myeloid compartment, previous studies show shifting of peripheral monocyte populations in PD patients as well as increased expression of chemokine receptor CCR2 on PD monocytes, suggesting enhanced migration of peripheral monocytes to the CNS.^38–40^ Rodent models of PD confirmed that activated, peripheral monocytes migrating and entering the CNS led to neurodegenerative outcomes.^31,41^ Analyses of the peripheral lymphoid compartment in PD demonstrate alterations in lymphocyte populations including CD4^+^ helper T cell, helper T-cell subsets, and CD8^+^ cytotoxic T cells.^42–46^ Additional studies describe a complex phenotype of the CD4^+^ compartment in PD patients with decreased anti-inflammatory subsets along with increased pro-inflammatory subsets.^47^ Multiple studies point to the direct involvement of T cells in response to pathogenic, aggregated species of a-syn. The abnormal protein causes a loss of peripheral immune tolerance with activation of multiple pro-inflammatory subsets of T cells.^17,18^ Evidence for pro-inflammatory T cells reactive to a-syn in mid to late stages of disease has been reported as has evidence that T-cell involvement is persistent in early, prodromal phases of disease.^16,19^

The immune system is a complex and tightly regulated network of innate and adaptive processes known to be compromised in aging and disease. Regulatory T cells (Tregs), an immunosuppressive T-cell subset, are critical regulators of immune cell tolerance and sustain immune homeostatic balance in health and disease. Treg dysfunction has been reported in multiple systemic and neurological disorders resulting in chronic and hyper activation of pro-inflammatory immune mechanisms.^35,48^ In neurodegenerative disease such as ALS and Alzheimer’s, dysfunction of Tregs is associated with significantly increased pro-inflammatory innate immune myeloid cells.^49–52^ In PD patients, Tregs are decreased in number and display impaired suppression of T-cell proliferation. In animal models of PD, restoring Tregs and their suppressive function reduces disease associated inflammation and provides neuroprotection.^17,53^

There is increasing evidence that substantial blood–brain barrier dysfunction, extensive neuro-immune cross talk, and infiltration of peripheral components contribute to the pathogenesis of PD.^54–57^ The present study adds to the evidence for alterations of the peripheral immune system. Peripheral myeloid and lymphoid cell populations become more pro-inflammatory as disease progresses. We document that PD Treg suppression of T-cell proliferation is significantly reduced. Most critically, we document that baseline Tregs do not suppress pro-inflammatory myeloid cells. Following ex vivo expansion, Treg suppression of T-cell proliferation in vitro is significantly enhanced, and suppression of pro-inflammatory myeloid cells is restored. Additionally, transcripts of *foxp3*, *il2ra* (CD25), *nt5e* (CD73), *il10*, *il13*, *ctla4, pdcd1* (PD1), and *gzmb* are increased in expanded Tregs, and each of these individually and in varying combinations have been implicated as contributing to the mechanisms whereby Tregs suppress proliferating T cells and activated pro-inflammatory myeloid cells.^58,59^ The overall evidence suggests that ex vivo expanded PD Tregs may provide a meaningful therapeutic option for ameliorating the immune dysfunction driving PD disease and progression.

## Results

### Activation-mediated shifts in PD peripheral monocyte populations

**Fig. 1:**
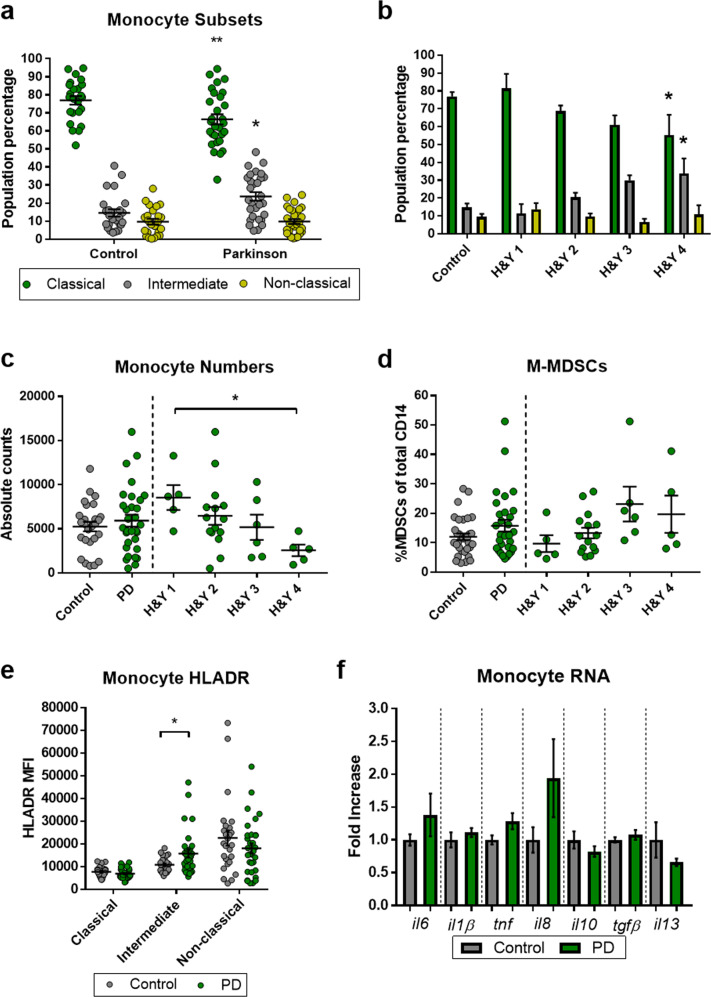
Peripheral monocyte populations are shifting and activating in PD. Analysis into the peripheral myeloid cells isolated from PD and controls was done to detect population and polarization changes due to disease and progression. **a** Flow cytometry analysis of shifting monocyte populations in control (*n* = 25) vs PD (*n* = 29). **b** Monocyte population shifts as a function of disease progression using the H&Y PD scale (C *n* = 25, H&Y1 *n* = 4, H&Y2 *n* = 16, H&Y3 *n* = 5, H&Y4 *n* = 4). **c** No change in overall monocyte counts in control vs PD analyzed samples but **d** decreasing monocyte numbers through disease progression using H&Y PD scale. **e** Trending increase in myeloid-derived suppressor cells in PD patients compared to controls (p = 0.09). **e** Flow cytometric analysis of pro-inflammatory, HLADR MFI signatures on cells in shifting monocyte populations (C *n* = 25, PD *n* = 29). **f** RNA analysis of pan monocytes isolated via negative, bead/column-based methods show increased trends in increased pro-inflammatory transcripts in monocytes from PD patients (*il6, il1β, tnf, and il8*) and decreased anti-inflammatory transcripts (*il10, tgfβ, and il13)* (C *n* = 12, PD *n* = 12). Numbers shown as averages ± SEM with Student’s *t* test or one-way ANOVA with appropriate post hoc testing for multiple comparisons as appropriate. *P*-values are *p < 0.05, **p < 0.01, and ***p < 0.001.

Peripherally derived myeloid cells are of increasing interest in PD disease and progression. An examination into peripheral myeloid cells was done via flow cytometric analysis following staining of whole blood from PD patients and controls (Supplementary Fig. 1). Classical monocyte populations (CD14 + CD16-) in PD were decreased along with an increase in the intermediate population (CD14 + CD16 + ) (Fig. 1a). There were no changes in the non-classical monocyte populations (CD14lowCD16 + ). However, in more advanced stages of disease, there was a shift from the classical monocyte population to the intermediate population (Fig. 1b). The total number of monocytes did not change in PD compared with controls but total monocytes decreased with later stages of disease (Fig. 1c). Myeloid-derived suppressor cells (MDSCs) are typically activated and increase their population numbers during chronic, systemic pro-inflammatory activation states. Flow cytometric analysis of monocytic MDSCs (CD14 + HLADR- CD11b + CD33 + ) revealed a slight increase in PD patients compared to controls with a trending increase in the later stages of disease (Fig. 1d). Next, we examined the HLA-DR inflammatory surface signature of these peripheral monocyte subsets in PD and control as surface expression of HLA-DR is upregulated during myeloid activation to promote pro-inflammatory signaling and communicate to T cells. HLA-DR mean fluorescent intensity (MFI) was increased in the PD intermediate monocyte population (Fig. 1e) but with no apparent deviation as PD patients progressed through disease. Monocyte inflammatory transcript analysis revealed trending increases of pro-inflammatory RNA transcripts from PD monocytes accompanied by decreasing signatures in known anti-inflammatory transcripts but did not reach significance (Fig. 1g). Specifically, we observed trending increases in *il6*, *tnf*, and *il8* along with decreases in *il10* and *il13*. Together these data suggest a disease-associated, pro-inflammatory shifting of the peripheral monocyte compartment in PD.

### Pro-inflammatory T-cell changes in PD

**Fig. 2:**
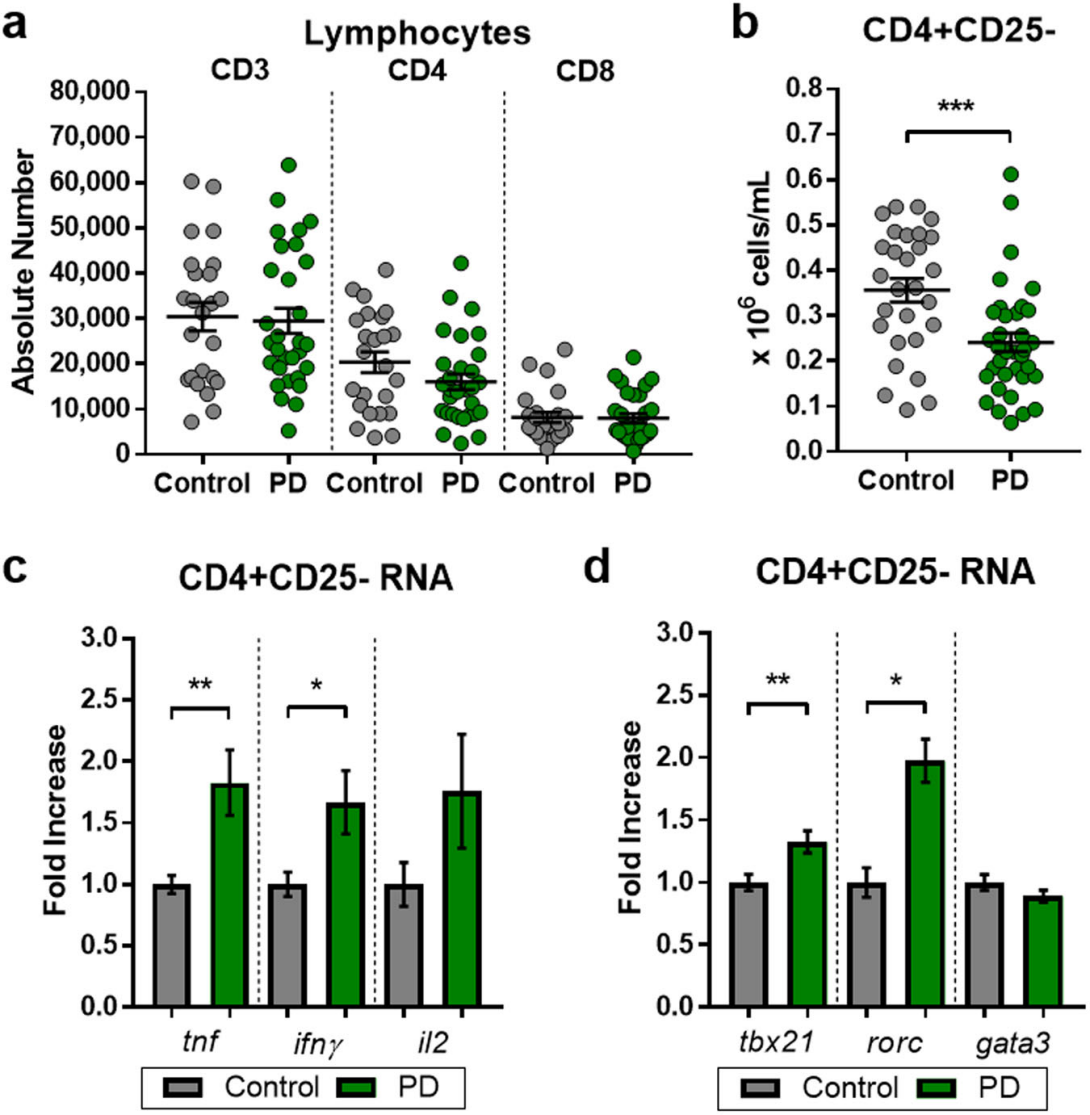
Peripheral T-cell changes in PD. An investigation into the complementary T-cell compartment of PD patients and controls to find pro-inflammatory changes was done. **a** Analysis of lymphocyte populations in blood samples from control and PD patients show no discernable difference in the numbers of CD3, CD4, or CD8 cell populations following flow cytometry (C *n* = 24, PD *n* = 30). **b** After bead/column-based isolation methods from peripheral blood, the number of T effector cells (CD4 + CD25-) is decreased in PD patients compared with controls (C *n* = 28, PD *n* = 35). Functional analysis of T effector polarization was done following isolation using RNA transcript analysis of **c** pro-inflammatory cytokine transcripts, *tnf, ifnγ*, and *il2*, and **d** RNA of transcription factors for T-cell polarization, *tbx21, rorc*, and *gata3* (C *n* = 16, PD *n* = 17). Numbers shown as averages ± SEM with Student’s *t* test or one-way ANOVA with appropriate post hoc testing for multiple comparisons as appropriate. *P*-values are *p < 0.05, **p < 0.01, and ***p < 0.001.

The interconnected and disease-associated compartment of T-cell populations in the periphery warrants increased investigation. T-cell populations from patients with PD and controls were examined by flow cytometry and RNA gene expression analysis following isolation from peripheral blood. There were no changes in the numbers of CD3^+^, CD4^+^, and CD8^+^ T cells from patients with PD compared with controls (Fig. 2a). Phenotypes and functions of T cells for patients with PD and controls were analyzed. A reduction was observed in the T effector (Teff) cell population (CD4 + CD25-) in PD samples (Fig. 2b). Following RNA isolation and RT-PCR analysis, the Teff population from patients with PD was polarized to a pro-inflammatory state as indicated by increased levels of pro-inflammatory transcripts *tnf*, *ifnγ*, and *il2* (Fig. 2c). *tbx21* and *rorc*, master transcription factors for T helper type 1 (Th1) and T helper 17 (Th17), respectively, were increased in patients with PD compared to controls (Fig. 2d). Our analysis of T-cell populations shows significant alterations consistent with a pro-inflammatory shift in PD patients.

### Treg numbers and function are impaired in PD patients

**Fig. 3:**
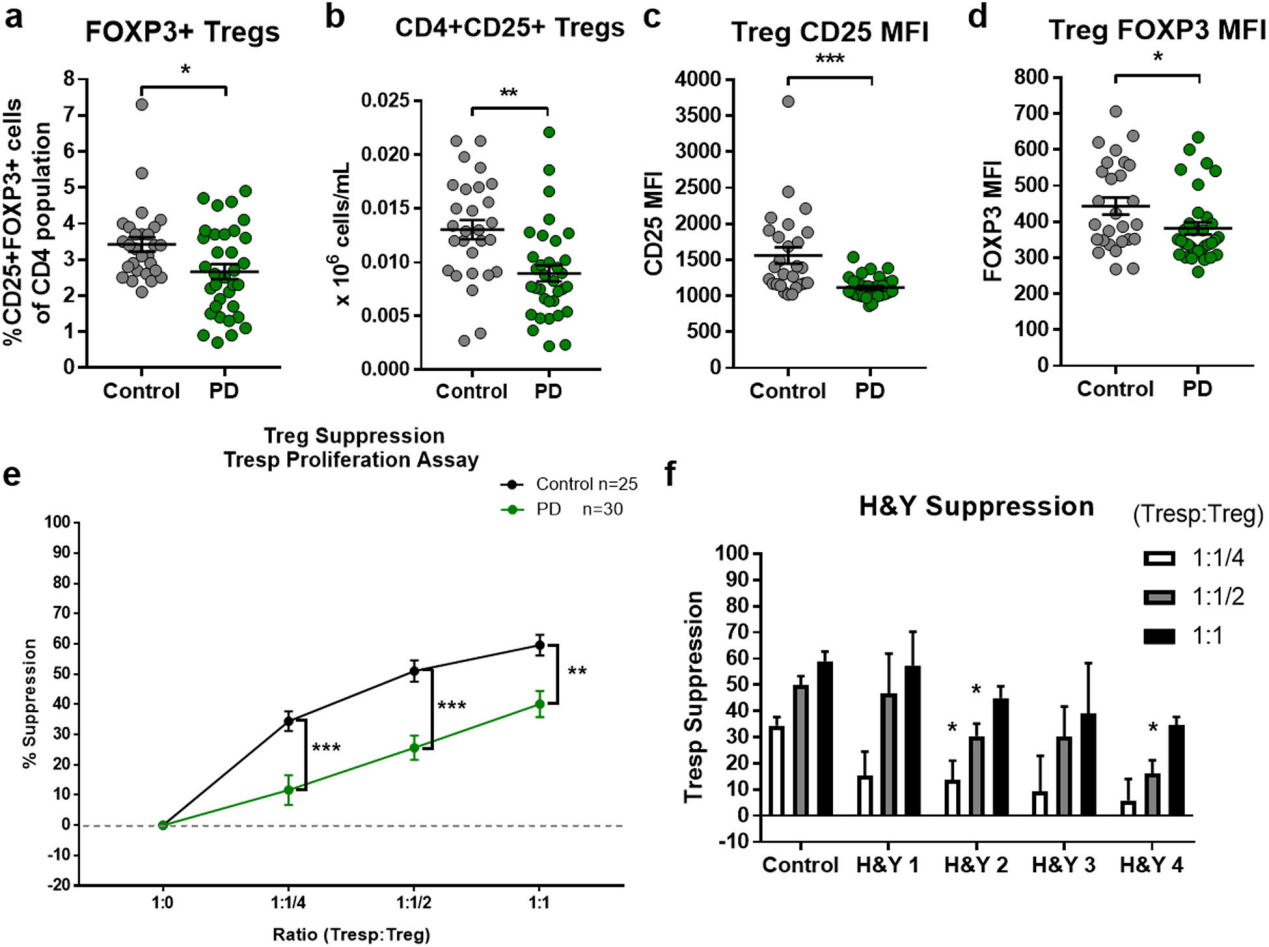
Investigation into PD Treg populations shows reduced numbers and impaired function. The number of Tregs in PD patients are decreased via **a** flow cytometric analysis of CD4 + CD25 + FOXP3 + cells as a percent of total CD4^+^ population and **b** counting of viable CD4 + CD25 + immune cells following bead/column-based isolation methods from peripheral blood (C *n* = 28, PD *n* = 34). Treg health and function markers were deceased in PD patients compared to controls when examining **c** CD25 protein MFI and **d** FOXP3 protein MFI from CD4 + CD25 + FOXP3 + cells during flow cytometry (C *n* = 28, PD *n* = 34). **e** Treg suppression of Tresp proliferation is impaired in PD patients compared to controls at ratios (Tresp:Treg) of 1:1, 1:1/2, and 1:1/4 (C *n* = 25, PD *n* = 30). **f** The suppressive capacity of PD Tregs on Tresp proliferation decreases with increasing PD disease burden using the H&Y disease scale (C *n* = 25, H&Y1 *n* = 4, H&Y2 *n* = 17, H&Y3 *n* = 5, H&Y4 *n* = 4). Numbers shown as averages ± SEM with Student’s *t* test or one-way ANOVA with appropriate post hoc testing for multiple comparisons as appropriate. *P*-values are *p < 0.05, **p < 0.01, and ***p < 0.001.

Immunosuppressive Tregs organize and orchestrate peripheral immune tolerance necessary to maintain inflammatory homeostasis while dysregulation in Treg numbers and/or function can lead to inflammation and disease. We analyzed the PD and control Treg populations, defined as CD4 + CD25 + FOXP3 + T cells, via flow cytometric analysis using peripheral whole blood samples. The analysis showed a significant decline in the number of Treg cells (% CD4^+^ T-cell population) in PD patients (2.67%) compared to controls (3.42%) (Fig. 3a). Corroborating this, a significant reduction in PD Tregs compared to controls was observed following Treg isolations from the blood (Fig. 3b). Additionally, the MFI analysis demonstrated that Tregs from patients with PD have reduced protein expression of CD25 and FOXP3 protein per cell (Fig. 3c, d). The lower levels of CD25 and FOXP3 suggest an alteration of the suppressive functions of PD Tregs, and prompted a direct assay of their suppressive functions. Isolated PD Tregs demonstrated profound dysfunction as they presented with severely reduced suppressive capacity compared to control Tregs (Fig. 3e). Control Tregs showed suppression of 59.6% (1:1 Tresp:Treg), 51% (1:1/2), and 34.4% (1:1/4) with corresponding patient Tresp proliferation assays while the PD Tregs could only suppress by 40% (1:1), 25.6% (1:1/2), and 11.6% (1:1/4). These data display an overall reduction in suppressive capacity of PD Tregs by approximately 22.5%. Additionally, PD Treg suppression seemed to worsen with increasing burden of disease (Fig. 3f). Heterogeneity was seen in both control and PD Tresp proliferation while no significance between the two or through disease progression was observed (Supplementary Fig. 5a). No correlation was seen between control or PD Treg suppression and Tresp proliferation capacity (Supplementary Fig. 5b). In our study, the PD patients demonstrated reduced circulating Tregs, aberrant markers of signaling, and reduced suppressive capacity on Tresp proliferation.

### Declining PD Treg function correlates with increasing pro-inflammatory T-cell activation

**Fig. 4:**
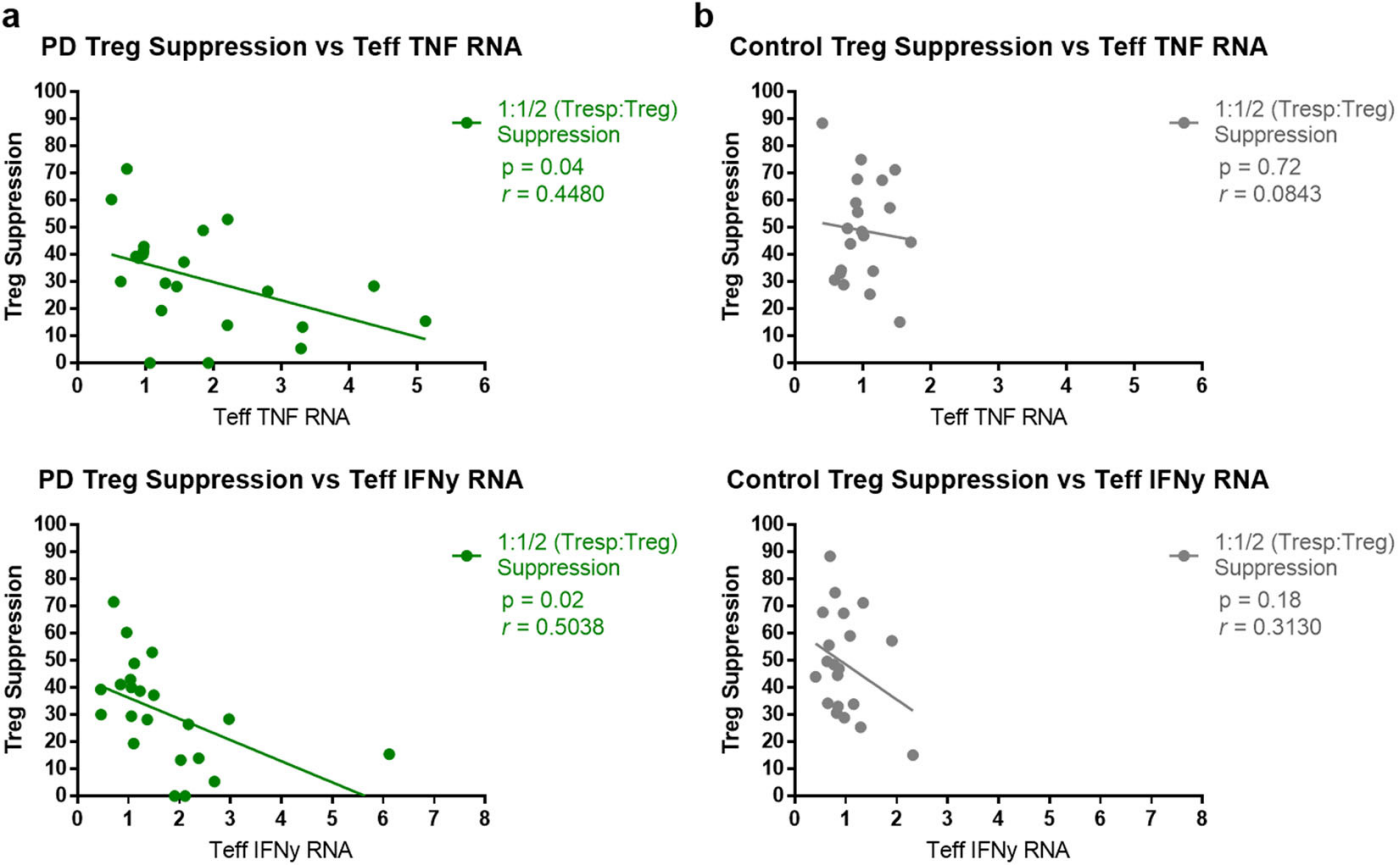
Analysis of Treg suppression and pro-inflammatory Teff transcripts. **a** A decrease in PD Treg suppression correlates with an increase in Teff-derived pro-inflammatory TNF transcripts. *r* = 0.4480 and *p* = 0.04 for PD Teff TNF transcripts against 1:1/2 (Tresp:Treg) ratio suppression assay. An increase in Teff IFNγ transcripts is correlated with declines in corresponding patient Treg suppression. *r* = 0.5038 and *p* = 0.02 for PD Teff IFNγ transcripts against 1:1/2 ratio (Tresp:Treg) suppression assay. **b** No significant correlation was seen between control Treg suppression and their corresponding Teff pro-inflammatory transcripts.

The loss of immune tolerance by Tregs can directly result in the subsequent increase in pro-inflammatory signaling by other immune cell populations and we examined this relationship in our PD patients. Treg suppression of T-cell proliferation significantly correlated with peripheral pro-inflammatory immune cell phenotypes. A decrease in PD Treg suppressive function at the 1:1/2 (Tresp:Treg) ratio correlated (*r* = 0.4480 *p* = 0.04) with an increase in *tnf* transcripts from corresponding PD isolated Teffs. Additionally, this decrease in PD Treg suppression also correlated (*r* = 0.4759, *p* = 0.03; *r* = 0.5038, *p* = 0.04) with an increase in PD Teff *ifnγ* transcripts (Fig. 4a). These correlations were not significant between control Treg suppression and their corresponding Teff pro-inflammatory transcripts (Fig. 4b).

### Treg ex vivo expansion restores Treg suppressive functions

**Fig. 5:**
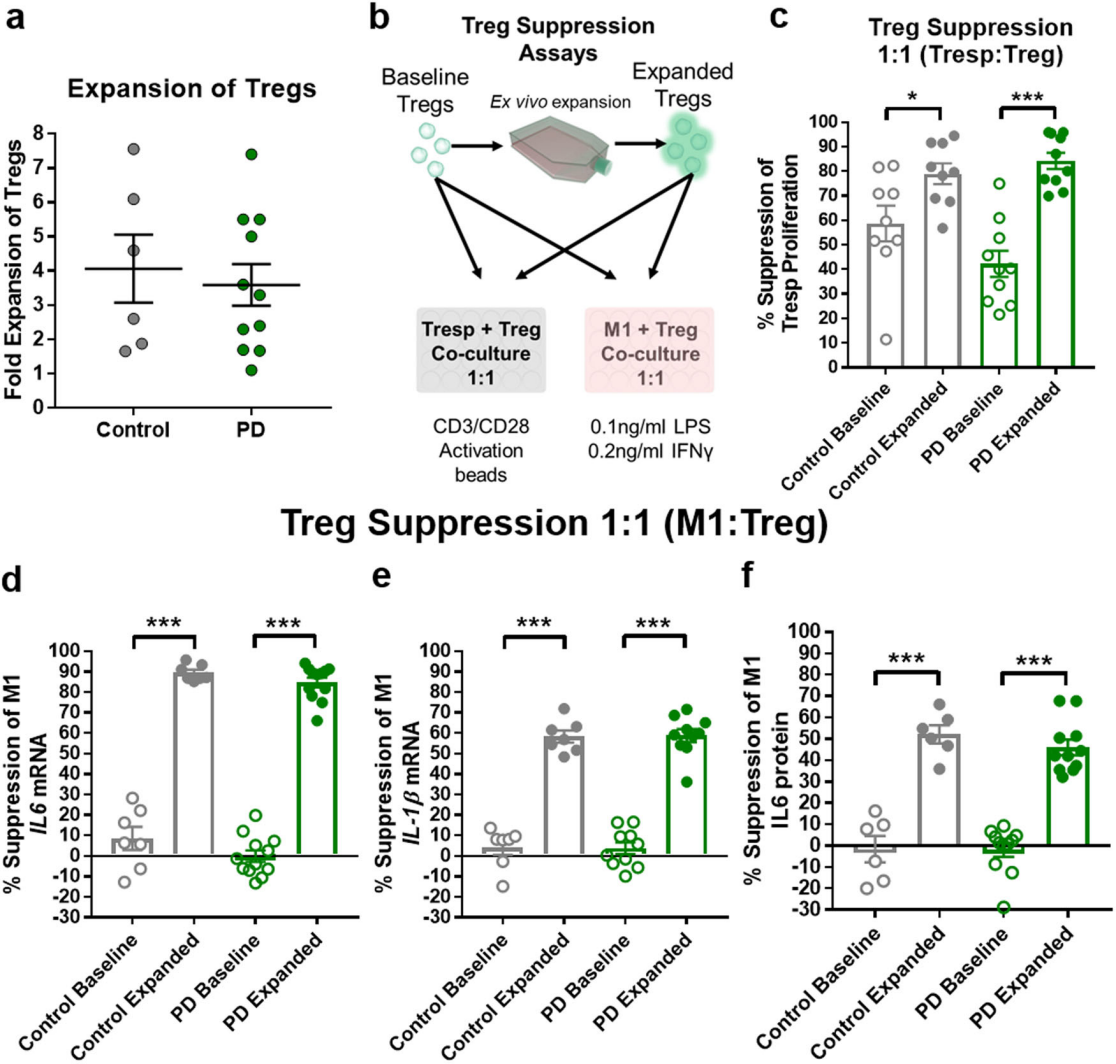
Tregs respond robustly to ex vivo expansion protocols generating increased Treg suppression of Tresp proliferation and pro-inflammatory myeloid cells. **a** Tregs from both control and PD patients expand their numbers following ex vivo expansion protocols. **b** Co-culture assays utilized to assess baseline and expanded Treg suppressive function. Baseline Tregs from control and PD patients are co-cultured with Tresp cells at 1:1 ratio for 5 days and proliferation assessed via tritium incorporation (C *n* = 9, PD *n* = 10). Independently and concurrently, baseline Tregs are co-cultured at 1:1 ratio with iPSC-derived pro-inflammatory myeloid cells overnight for 18 h following stimulation with LPS and IFN-γ. Suppression of pro-inflammatory myeloid effector function measured by *il6 and il1β* RNA transcripts and IL-6 protein from co-culture media. Control and PD Tregs will be ex vivo expanded and subjected to the same battery of co-culture suppression tests to examine increases in suppressive function following expansion protocols. **c** Increased suppression of Tresp proliferation following ex vivo expansion protocols of both control and PD Tregs. **d**–**f** Robust increase in suppression of *il6* transcript, *il1β* transcript, and IL-6 protein following 18 h co-culture of expanded Tregs with iPSC-derived pro-inflammatory myeloid cells (C *n* = 6–7, PD *n* = 10–12). Numbers shown as averages ± SEM with Student’s *t* test or one-way ANOVA with appropriate post hoc testing for multiple comparisons as appropriate. *P*-values are *p < 0.05, **p < 0.01, and ***p < 0.001.

Although PD Tregs show reduced number and extensive dysfunction, we investigated their ability to respond to protocols designed to increase their immunosuppressive function and tested them in co-culture with proliferating T cells and pro-inflammatory myeloid cells. Following ex vivo culture, Tregs from patients with PD and controls expanded to a similar variable extent (Fig. 5a). Tregs from patients and controls were co-cultured with responder T cells (Tresps) or pro-inflammatory iPSC-derived myeloid cells (Fig. 5b). Co-culture of control Tregs/Tresps had a 59% suppressive capacity at baseline that increased to 79% suppression following expansion. For PD Tregs, baseline suppression levels of Tresps were 42% and were increased to 84% following expansion (Fig. 5c). Baseline Tregs from neither PD patients nor controls suppressed RNA transcription of pro-inflammatory cytokines *il6 or il1β*, from iPSC-derived pro-inflammatory myeloid cells (Fig. 5d, e). Following ex-vivo expansion, both PD and control Tregs were clearly able to suppress iPSC-derived pro-inflammatory myeloid cells. Expanded PD and control Tregs suppressed *il6* transcription by 85 and 89%, respectively (Fig. 5d), and suppressed *il1β* by 59 and 58%, respectively (Fig. 5e). Supernatants from the co-culture assays were collected and examined for levels of IL-6 protein. As with the RNA transcript data, baseline IL-6 protein suppression of PD and control Tregs was minimal with no suppressive capacity (Fig. 5f). However, following expansion, PD and control Tregs suppressed IL-6 protein production and release by 46 and 52%, respectively. These data document that PD and control Tregs respond robustly to the ex vivo expansion protocols in both number and suppressive function, and exceed baseline suppression levels.

### Gene expression profiles of PD and control Tregs at baseline and following expansion

**Fig. 6:**
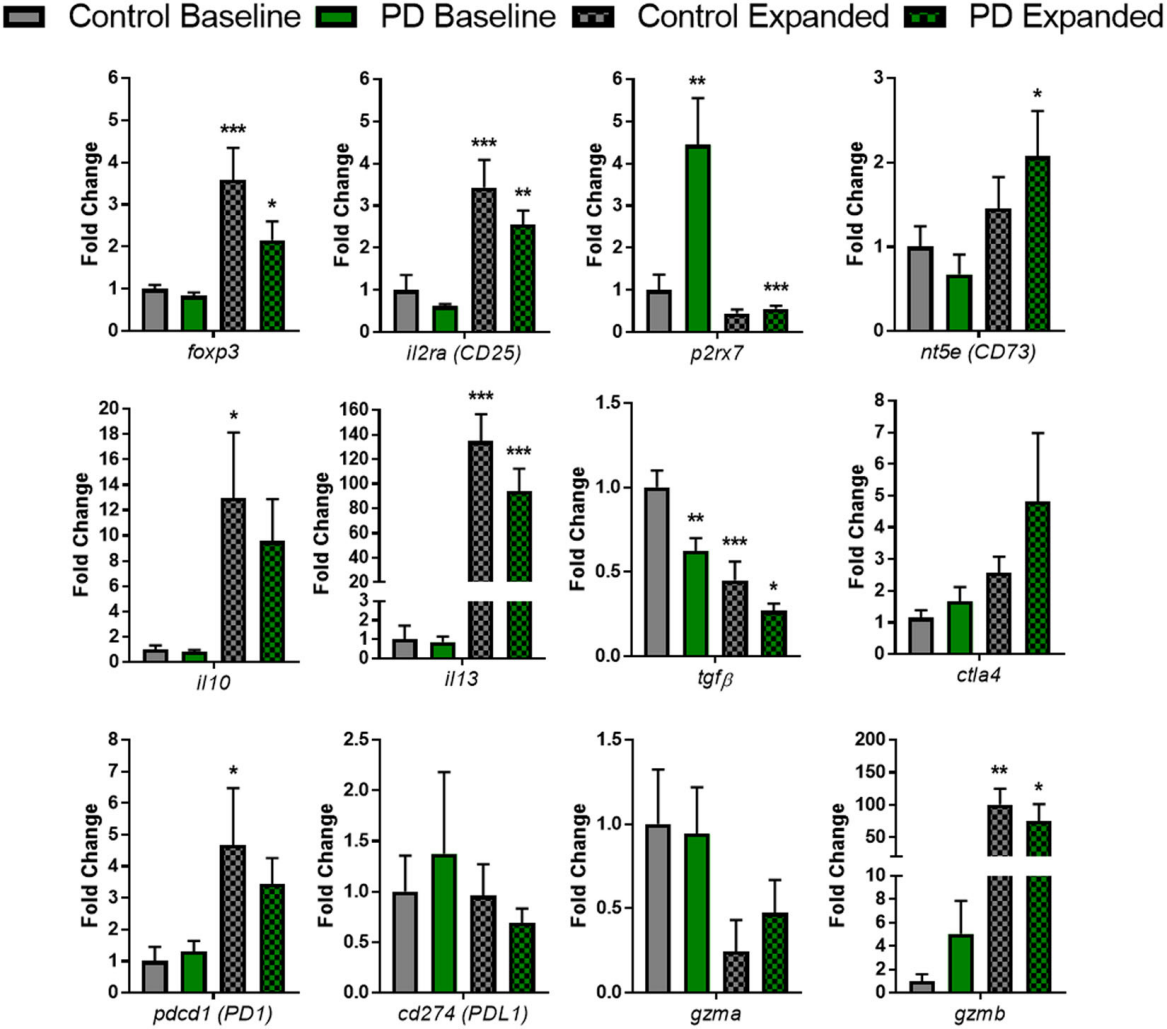
Transcript analysis of expanded Tregs demonstrate enhancements of Treg anti-inflammatory and immunosuppressive mechanisms. RNA transcript analysis of markers of Treg health, suppressive function, and other mechanisms of Treg anti-inflammatory and immunomodulatory function. Increased levels of Treg health markers following expansion of *foxp3* and *il2ra* (CD25). *p2rx7*, a marker increased with Treg dysfunction, is increased in PD patients compared to controls at baseline and is reduced following expansion protocols. Markers of anti-inflammatory function, *nt5e* (CD73) *il10, and il13* are significantly increased in control and PD expanded Tregs. There is a decrease in *tgfβ* in PD Tregs compared to control at baseline and those transcripts are further reduced following expansion protocols. Additional transcript markers of Treg function are increased post expansion in Tregs such as *ctla4*, *pdcd1* (PD1), and *gzmb* while other markers are trending toward decreasing levels following expansion such as *cd274* (PDL1) and *gzma* (C *n* = 6–8, PD *n* = 6–8). Numbers shown as averages ± SEM with one-way ANOVA with appropriate post hoc testing for multiple comparisons as appropriate. *P*-values are *p < 0.05, **p < 0.01, and ***p < 0.001.

The mechanisms of enhanced Treg suppression following expansion are of interest for therapeutic opportunity and disease-modifying means. We performed transcript analysis from RNA isolated from both PD and control Tregs at baseline and after ex vivo expansion (Fig. 6). *foxp3* and *il2ra* (CD25) transcripts were both increased in PD and control Tregs. The expression of *p2rx7* was increased in PD baseline Tregs compared with control Tregs, possibly suggesting a mechanism of dysfunction and inactivation in this population of Tregs during PD. Interestingly, PD Tregs had reduced expression of *p2rx7* following expansion. Although no differences were observed in Treg expression profiles of *nt5e* (CD73), *il10*, and *il13* between PD and control Tregs at baseline, expression of both *nt5e* and *il13* was increased following expansion. It is important to note that the levels of *il13* were increased by 94 and 134 fold in the expanded PD and control Tregs, respectively. Endogenous levels of *tgfβ* were decreased in baseline PD Tregs compared to baseline controls. Interestingly, both PD and control Tregs reduced their *tgfβ* expression following expansion. Examination of transcripts involved in other cited mechanisms for Treg suppression such as *ctla4*, *pdcd1* (PD1), *cd274* (PDL1), and *gzma* were analyzed. After ex vivo expansion, we noted a trend toward increased levels of *ctla4* and *pdcd1* along with decreased trends in *cd274* and *gzma*. Similar to the remarkable increase in levels of *il13* following expansion, we noted a similar pattern with *gzmb* whereby the expanded control Tregs were increased 101 fold compared to baseline and expanded PD Tregs were increased 75 fold. Collectively, these data suggest that ex vivo expansion of Tregs, whether from diseased or control, generates Treg cells enriched with anti-inflammatory and immunosuppressive transcripts.

## Discussion

Central nervous system (CNS) inflammation is known to play a prominent role in the pathobiology of PD. However, peripheral inflammation has not been well studied in patients with PD. The current study found that subsets of immune cells in the periphery were pro-inflammatory in patients with PD; there were also increases in many cytokine transcripts that trended in the pro-inflammatory direction in these patients. Furthermore, and most significantly, PD Tregs were dysfunctional and their numbers decreased in patients with PD which may compound these pro-inflammatory responses. Finally, this study demonstrates that dysfunctional PD Tregs were reversed following ex vivo expansion; the expanded Tregs were immunosuppressive on T-cell proliferation assays and suppressed myeloid cell pro-inflammatory signaling. Phenotyping the expanded Tregs via RNA transcript analysis documented the altered Treg profiles following expansion. Thus, this study demonstrates that in addition to the well-known CNS pro-inflammatory responses in patients with PD, there are novel concurrent peripheral pro-inflammatory responses in PD that reflect burden of disease.

The peripheral immune compartment is of increasing interest in neurodegenerative diseases. Neuro-immune crosstalk results from extensive blood–brain barrier breakdown and infiltration of peripheral cells into the CNS. Many studies have shown that collateral damage from neuronal cell death as well as pathogenic species of a-syn promote pro-inflammatory activation of myeloid cells such as microglia and monocytes/macrophages, and also cause dysfunction of a wide array of central and peripheral immune constituents.^29,31,60,61^ The current study demonstrated that monocytes in the blood of patients with PD shifted from the classical monocyte subset into the intermediate subset, and this shift increased with escalating disease burden. The intermediate monocyte subset represents monocyte maturation and a shift to a more pro-inflammatory subset.^62–64^ A similar study of monocyte populations from patients with PD reported an enrichment in the classical monocyte subset, although a different regrouping of the three monocyte populations combining the classical and intermediate groups allows limited comparisons between both studies.^40^ The flow cytometric analysis of monocyte HLA-DR surface expression, a MHC-class II receptor upregulated in monocyte maturation and activation, revealed increased HLA-DR in PD intermediate monocytes. Disease progression was not associated with increased intermediate monocyte HLADR cell expression. Although they did not reach significance, there was a trend for increased pro-inflammatory gene expression in these cells; *il6, tnf*, and *il8* levels were up, whereas *il10 and il13* levels were down. Another group showed that PD monocytes were altered in vitro along with a higher proliferative capacity.^65^ The current study demonstrated that the total number of monocytes were unchanged in patients with PD which is in accordance with other reports.^40,66^ There were increased MDSCs in the periphery of patients with PD. Increases in MDSCs are prevalent in cancer, chronic inflammatory disorders, and reported recently in early stages of neurodegenerative diseases such as Alzheimer’s and Parkinson’s disease.^51,67^ While not significant, this increase in suppressive MDSC populations in PD patients can dilute the pro-inflammatory phenotype analysis of the isolated, total monocyte population that we analyzed. This MDSC population increased incrementally as disease progressed in PD patients—possibly as an effort to counterbalance the loss of suppressive Tregs, or alternatively due to an increase of a dysfunctional or pro-inflammatory MDSC population. More advanced, extended analyses of the peripheral myeloid compartment and their phenotypes in patients with PD will allow us to understand their respective contribution to PD while also being a potential proxy for the microglial activation states during disease progression.

Early post-mortem analysis of CNS tissue from patients with PD showed diffuse infiltration of activated T cells in areas with microglial activation and alpha-synuclein accumulation. Previous studies examining peripheral blood-derived T-cell changes were inconsistent and less than definitive.^42,43,46,68,69^ The current study documented no changes in the CD3^+^ and CD8^+^ T-cell populations in patients with PD; there was a trend for less CD4^+^ cells in these patients. Peripheral populations of Teffs were significantly decreased in PD, possibly due to trafficking to the CNS.^11,13,70^ Interestingly, the decreased population of Teffs express increased pro-inflammatory transcripts such as *tnf*, *ifnγ, rorc*, and *tbx21*. These signatures are indicative of a Th1 and Th17 pro-inflammatory polarization of T cells. The observation that there was a Th1 bias in patients with PD was previously reported.^47^ Th17 cells, another pro-inflammatory T-cell subset that produces IL-17, has been reported to be increased in the periphery of PD patients and can directly induce IL-17R-mediated midbrain neuronal cell death in an in vitro co-culture model of PD.^67,71^ Of significance, it is documented that PD T cells recognize and respond to alpha-synuclein epitopes which can drive T-cell responses.^16,19^ The correlations of PD Teff pro-inflammatory *tnf* and *ifnγ* RNA against Treg produced anti-inflammatory *tgfβ* and *il10* transcripts proved non-significant (data not shown). Previous reports of increased Th17 cells in the periphery of PD patients prompted us to examine associations of Th17 cells and Treg cells in the balance of peripheral immune tolerance. We did not find a correlation between Treg cells and the expression of Teff *rorc*, the precursor transcript for the Th17-polarizing transcription factor (Supplementary Fig. 4). Future experiments analyzing these sub-populations of CD4 + Teff and Treg cells are warranted to answer these questions. The associations between Teff inflammatory output and Treg function could lead to significant targets for preventing or ameliorating Treg dysfunction in disease. Together, these data support the finding that pro-inflammatory polarization is occurring in T cells of PD patients.

Tregs are immunosuppressive cells that regulate the immune response and maintain homeostasis in the inflammatory microenvironment. Extensive studies of Tregs and their functional involvement in health and disease have led to the development of therapies that ameliorate disease.^49,72–74^ Tregs are found to be decreased and/or dysfunctional in a number of diseases such as systemic lupus erythematosus, type 1 diabetes, multiple sclerosis, amyotrophic lateral sclerosis, and Alzheimer’s disease.^49,50,72,75–82^ The current study demonstrates decreased numbers of Tregs in patients with PD. More importantly, this study demonstrates impaired Treg suppressive function starting early in disease and worsening as disease progresses. CD25 and FOXP3 proteins were also shown to be decreased on PD Tregs which could suggest increased cell dysfunction or disruption of necessary developmental or maintenance signaling for Tregs. Future investigations into diseased Tregs are planned to analyze these aberrant baseline signaling pathways that could lead to deleterious Treg issues, increased peripheral inflammation, and subsequent initiation of neurodegenerative processes. Previous studies reported mixed results concerning Treg numbers and their suppressive functions in patients with PD.^42,69,83,84^ In animal models of PD, Tregs were found to be dysregulated^85^; Treg depletion results in exacerbation of inflammation and neurodegeneration.^86^ Therapies harnessing Tregs by reconstitution or functional restoration attenuated neuroinflammation and subsequently slowed neurodegeneration.^17,87,88^ Dopamine has been hypothesized to suppress Treg function in vitro, and dopamine replacement therapy has been thereby proposed to be a potential confounding variable in Treg dysfunction in PD.^89^ However, another study examining Tregs in dopamine-naïve and dopamine-treated PD patients demonstrated no change in Treg numbers and suppressive function.^47^ We found that addition of dopamine at both 1 and 0.1 uM concentrations had no effect on control or PD Treg suppressive function on Tresp proliferation in vitro (Supplementary Fig. 6).

The correlations in this study found that the increasing PD Treg dysfunction resulted in an increase in pro-inflammatory activation of PD Teff cells. It is possible that a pro-inflammatory milieu of specific signaling components such as IL-6, IFNγ, TNF, etc. could be driving Treg dysfunction. Investigations into causative peripheral factors causing Treg dysfunction is ongoing, but our preliminary data suggest that these cytokines, per se, do not cause Treg dysfunction in vitro, and direct contact may be a relevant factor. Additionally, targeting dysfunctional Tregs directly may provide a promising route to suppress the inflammatory cascades in PD. Several therapeutic paradigms currently exist that target Tregs to improve their numbers and function. Stimulating IL-2 receptor along with inhibiting the mTOR pathway present promising avenues for correcting dysfunctional Tregs in vivo.^90^ This strategy works in a neutral setting but disease complicates the viability of this strategy as the pro-inflammatory milieu provides large thresholds to overcome while cells may be unresponsive to the therapy due to dysfunction caused by chronic inflammation. Previous preliminary clinical studies in ALS patients demonstrated that Tregs appeared unresponsive to in vivo-targeted therapies to restore suppressive function; however, a phase 2a clinical trial from a European consortium suggests that subcutaneously administered IL-2 may enhance Tregs in vivo in ALS, and potentially provide clinical benefit.^73^ With relevance to PD, Treg targeted therapy utilizing recombinant GMCSF to stimulate Tregs in vivo proved efficacious in inducing Treg responses and protecting against nigrostriatal degeneration in pre-clinical models of disease.^88,91–93^ Subsequently, a phase 1 clinical trial using this therapy in a small PD population demonstrated that the therapy was safe and well tolerated and warrants further investigation.^94^ An alternative approach to enhancing Tregs is to isolate and purify Tregs from peripheral blood, expand them in vitro, and administer autologous infusions of expanded Tregs as a cell-based therapeutic.^74^ This is a promising strategy currently underway in ALS. Our present data in PD demonstrate that ex vivo expansion not only restores suppressive function but also significantly enhances the suppressive function beyond typical baseline levels. The expanded Tregs potently suppress multiple pro-inflammatory pathways in myeloid cells and inhibit Tresp proliferation. This study provides evidence that these expanded Tregs would be effective in targeting and suppressing both innate and adaptive immune processes that are altered and pro-inflammatory in PD.

The mechanisms by which expanded Tregs induce significant suppression are not fully understood. Following expansion of control and PD Tregs, we noted increased expression of Treg health and function markers, *foxp3* and *il2ra* (CD25). These gene signatures demonstrate a traditional Treg gene signature that is enhanced following the expansion process. We have found, previously and in this study, that the Treg expansion procedures produce a consistent flow signature of cells enriched with the Treg markers CD4 + CD25 + FOXP3 + (Supplementary Fig. 3). We also observed increased anti-inflammatory cytokine transcripts, *il10* and *il13*, and increased CD73 transcript, *nt5e*, which can mediate inflammatory cell suppression by converting AMP to adenosine. Additionally, *gzmb* was increased following expansion in both control and PD Tregs, and *gzmb* provides an alternative mechanism of suppression i.e., inducing cell death. Collectively, these results, together with those that did not reach statistical significance, such as CTLA4 and PD1, suggest that expanded Tregs could use multiple mechanisms to mediate suppression, and such mechanisms could differ under different circumstances. There is increasing importance in understanding the mechanisms by which expanded Tregs are able to suppress both T cell and pro-inflammatory myeloid cells. Our data suggest that there are multiple mechanisms working synergistically, as suggested and outlined by Sakaguchi et al. and others.^58,59^

Overall, this study documents the shift of PD peripheral immune cells to pro-inflammatory phenotypes that increase with increasing burden of disease. PD Treg numbers and immunosuppressive functions are decreased, and correlate with the increased pro-inflammatory phenotypes. Furthermore, ex vivo expansion of dysfunctional PD Tregs restores their suppressive functions and enhances their ability to reduce Tresp proliferation and decrease pro-inflammatory myeloid cell responses. Expanded Tregs provide a potentially meaningful therapeutic strategy for cell-based immunomodulatory therapies for diseases with acute or chronic pro-inflammatory insults such as PD.

## Methods

### Patient recruitment for PD patients and controls

PD patients (*n* = 39, M/F: 27/12, age: 70.6 ± 8.4) and age-matched healthy controls (*n* = 31, M/F: 11/20, age: 69.5 ± 8.9) were recruited to the study by the Houston Methodist Neurological Institute under the direction and evaluation of Dr. Eugene C. Lai and his Neurodegenerative Disease Clinic. Patient assessment and diagnosis were evaluated using the Movement Disorder Society clinical diagnostic criteria for PD.^95^ Written informed consent was obtained from PD patients and controls according to approved protocols evaluated by the Houston Methodist Institutional Review Board (IRB). Motor phenotypes of PD patients were evaluated using the Hoehn and Yahr (H&Y) scale which describes the motor manifestations of the disease according to stages (H&Y 1→H&Y 5). Additional cross analyses of variables such as age and gender revealed no significant confounding effects in the data representing PD vs controls in this study (data not shown).

### Flow cytometry

Immune cell populations were analyzed from peripheral blood and in vitro paradigms with a BD Bioscience LSR II bench top flow cytometer using immune cell fluorescent probes. Probes used for myeloid analysis include eBioscience (Thermofisher/Invitrogen catalog #) anti-human: CD14-V450 (48-0149-42), CD16-FITC (11-0168-42), HLA-DR- PerCP-Cy5.5 (45-9956-42), CD33-APC (17-0338-42), and CD11b-PE (12-0118-42). Monocyte populations are classified as follows: Classical monocytes (HLADR + CD14 + CD16-), Intermediate monocytes (HLADR + CD14 + CD16 + ), Non-classical monocytes (HLADR + CD14low CD16 + ), and myeloid-derived suppressor cells (HLADR- CD14 + CD11b + CD33 + ) (Supplementary Fig. 1). Identification of MDSCs based on suggested identification standards and previous lab studies investigating MDSCs.^51,96,97^ Lymphocyte population utilized anti-human: CD3-AF700 (eBioscience: 56-0037-42), CD4-v500 (BD Biosciences: 560768), CD25-PerCP-Cy5.5 (BD Biosciences: 560503), FOXP3-AF488 (eBioscience: 53-4776-42), and CD8-v450 (eBioscience: 48-0088-42). Treg cells are classified as CD3 + CD4 + CD25 + FOXP3 + cells (Supplementary Fig. 2). Viable cells were stained using Live/Dead Fixable Blue Dead Cell Stain Kit (Invitrogen: L23105) and appropriate isotype control antibodies were utilized according to their respective fluorophore and company. For intracellular staining, cells were fixed and permeabilized using FoxP3/Transcription Factor Staining Buffer Set (BD 560098). Cell population descriptions can be found in Supplementary Table 1.

### Immune cell isolations

Immune cells were isolated from peripheral blood of PD patients and controls using a Lymphoprep (Stemcell) density gradient followed by positive or negative bead-based, magnetic column (Miltenyi Biotec) isolations. For peripheral monocytes, negative isolation of desired population was obtained using Human Pan Monocyte Isolation Kit (Miltenyi Biotec). Treg and Tresp populations were obtained from PBMC pool using the CD4 + CD25 + Regulatory T Cell Isolation Kit (Miltenyi Biotec) whereby Tresps were negatively isolated in the flow through while the Tregs were positively isolated and run through the column twice for purity.

### RNA purification and RT-PCR analysis

RNA was isolated from blood-isolated immune cells, in vitro experiments, and ex vivo expansions using Trizol reagent followed by Direct-zol RNA MiniPrep Plus Kit (Zymo Research). RNA concentration/quality were assessed using Nanodrop spectrophotometer and Quantitative RT-PCR (qRT-PCR) experiments were performed using a One-Step RT-PCR kit with SYBR Green and a Bio-Rad iQ5 Multicolor Real-Time PCR Detection System. Primers for the study were acquired from BioRad and the relative expression of each mRNA was calculated using the ΔΔCt method with normalization to β-actin and relative to control samples. Primer information can be found in Supplementary Table 2.

### Treg suppression of Tresp proliferation assays

Patient Tresp and corresponding patient Tregs were isolated and cultured in 96-well, round-bottom plates at a density of 5 × 10^4^ cells per well (in triplicate) at ratios of 1:1, 1:1/2, and 1:1/4 (Tresp:Tregs). A CD3/CD28 stimualtion reagent, Human Treg Suppression Inspector (Miltenyi Biotec), was added to the co-culture for five days followed by the addition of tritiated thymidine (Amersham Life Sciences) for 18 h. Proliferation is measured via tritium incorporation.

### Treg suppression of iPSC-derived, pro-inflammatory myeloid cells

Our lab has recapitulated published protocols for the generation of mature myeloid cells from an induced pluripotent stem cell (iPSC) source (Yanagimachi et al. 2013). Briefly, iPSCs are transformed in four culture steps to become floating CD14^+^ myeloid cells. CD14 cells are isolated from the supernatant using human CD14 Microbeads (Miltenyi Biotec) and magnetic columns. CD14 cells are then cultured for 7 days in 10% FBS 1640 media supplemented with GMCSF (50 ng/mL; Miltenyi Biotec) to generate resting myeloid cells (M0). We harvest M0 cells with enzyme-free/PBS-based cell dissociation buffer (Gibco) and plated at 5 × 10^4^ cells per well in a 24-well plate. M0 cells are allowed to settle for an hour followed by a stimulation using lipopolysaccharide (LPS) (0.1 ng/mL; Sigma) and gamma interferon (IFN-γ) (0.2 ng/mL; eBioscience) to create activated, pro-inflammatory myeloid cells (M1). Tregs are co-cultured at 1:1 ratio with M1’s overnight (~20 h) to assess suppression of myeloid-specific pro-inflammatory markers. Cultured Tregs and M1 cells are individually isolated for RNA transcript analysis and cultured media collected to assess cytokine protein levels via ELISA (Sigma).

### Treg ex vivo expansion protocols

Tregs are isolated via bead-based negative selection as described previously. Tregs are suspended at a concentration of 1 × 10^5^ cells per well in 100 uL tissue culture media supplemented with 100 nM rapamycin (Miltenyi Biotec), 500 IU/mL IL-2, and Dynabeads Human Treg Expander (Gibco) according to the manufacturer’s protocol. Fresh media mix with rapamycin and IL-2 were added to the cells every 2–3 days. After 10–14 days of culture, the Tregs were harvested, washed, and utilized in their respective suppressive function assay.

### Statistical analysis

Statistics were generated using Graphpad Prism software. Analysis of two groups was performed with Student’s *t* test while analyzing more than two groups utilized one-way ANOVA with appropriate multiple comparisons testing. Data are expressed as mean ± SEM and *p* values reported according to the New England Journal of Medicine suggested output and guidelines.

### Reporting summary

Further information on research design is available in the Nature Research Reporting Summary linked to this article.

## Supplementary information

Supplementary Information

Reporting Summary

## Data Availability

The data collected during this study are available from the corresponding author upon reasonable request from qualified individuals.
