# Supplementary material for: Ex vivo expansion of dysfunctional regulatory T lymphocytes restores suppressive function in Parkinson’s disease
Source: NPJ Parkinsons Dis. 2021 May 13;7:41. doi: 10.1038/s41531-021-00188-5 (PMC8119976; doi:10.1038/s41531-021-00188-5)
Supplement: Supplementary file 1 — Supplementary Information [file 41531_2021_188_MOESM1_ESM.docx]

| Cell Population | Marker combination |
| --- | --- |
| Classical monocytes | HLADR+ CD14+ CD16- |
| Intermediate monocytes | HLADR+ CD14+ CD16+ |
| Non-classical monocytes | HLADR+ CD14low CD16+ |
| Monocytic myeloid-derived suppressor cells | HLADR- CD14+ CD11b+ CD33+ |
| Regulatory T cells (Tregs) | CD3+ CD4+ CD25+ FOXP3+ |

**Supplementary Tables:**

**Supplementary Table 1**

Cell marker descriptions for important cell types analyzed via flow cytometry in PD patients and controls.

| PrimePCR SYBR Green Assay | Company | Unique Assay ID |
| --- | --- | --- |
| actb | Bio-Rad | qHsaCED0036269 |
| il6 | Bio-Rad | qHsaCID0020314 |
| il1β | Bio-Rad | qHsaCID0022272 |
| tnf | Bio-Rad | qHsaCED0037461 |
| il8 | Bio-Rad | qHsaCED0046633 |
| il10 | Bio-Rad | qHsaCED0044704 |
| tgfβ | Bio-Rad | qHsaCID0017026 |
| il13 | Bio-Rad | qHsaCID0020181 |
| ifnγ | Bio-Rad | qHsaCED0043378 |
| il2 | Bio-Rad | qHsaCID0015409 |
| tbx21 | Bio-Rad | qHsaCID0006532 |
| rorc | Bio-Rad | qHsaCID0008528 |
| gata3 | Bio-Rad | qHsaCID0017793 |
| foxp3 | Bio-Rad | qHsaCID0007630 |
| il2ra | Bio-Rad | qHsaCID0012553 |
| p2rx7 | Bio-Rad | qHsaCID0012839 |
| nt5e | Bio-Rad | qHsaCID0036556 |
| ctla4 | Bio-Rad | qHsaCED0003794 |
| pdcd1 | Bio-Rad | qHsaCID0014211 |
| cd274 | Bio-Rad | qHsaCID0036468 |
| gzma | Bio-Rad | qHsaCID0008274 |
| gzmb | Bio-Rad | qHsaCID0013766 |

**Supplementary Table 2**

Information on primer sets and availability of PCR primers utilized in the study. Detailed primer information can be found using the unique assay ID when inquiring with the Bio-Rad primer website.

**Supplementary** **Figures:**


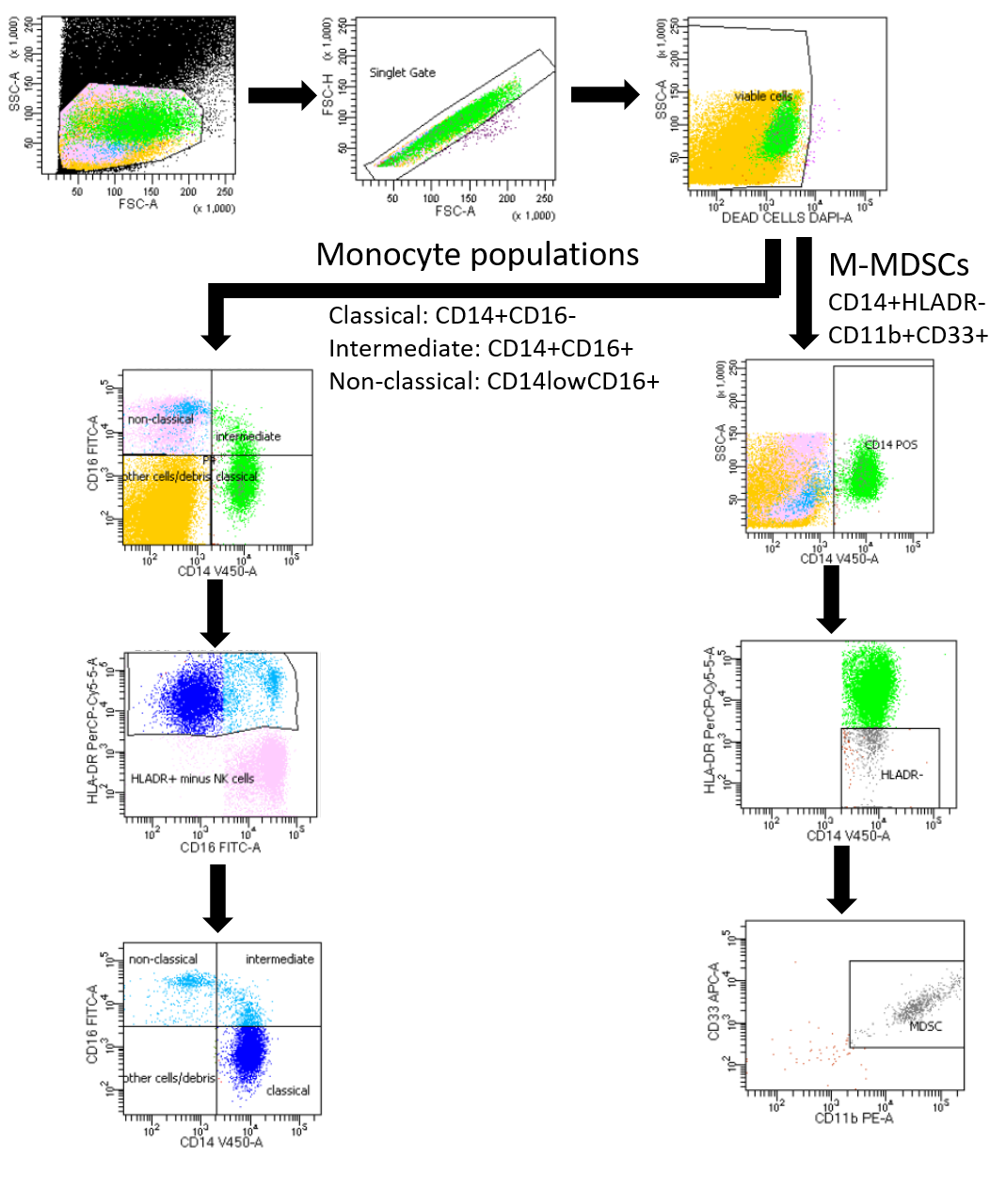


**Supplementary** **Figure 1**

Myeloid flow cytometry gating paradigm. Flow cytometry gating paradigm for the analysis of monocytes and monocytic myeloid-derived suppressor cells (M-MDSCs) from control and PD patients. Peripheral blood is isolated from control and PD patients and stained with the following assortment of cell markers: Live cells (DAPI), CD14, CD16, HLA-DR, CD33, and CD11b. Monocytes are defined as being live, single cells that express HLA-DR signature and also CD14 and/or CD16 (Classical: CD14+CD16-, Intermediate: CD14+CD16+, Non-classical: CD14lowCD16+). M-MDSCs are classified as live, single cells that are CD14+ HLA-DR- CD11b+CD33+.

**
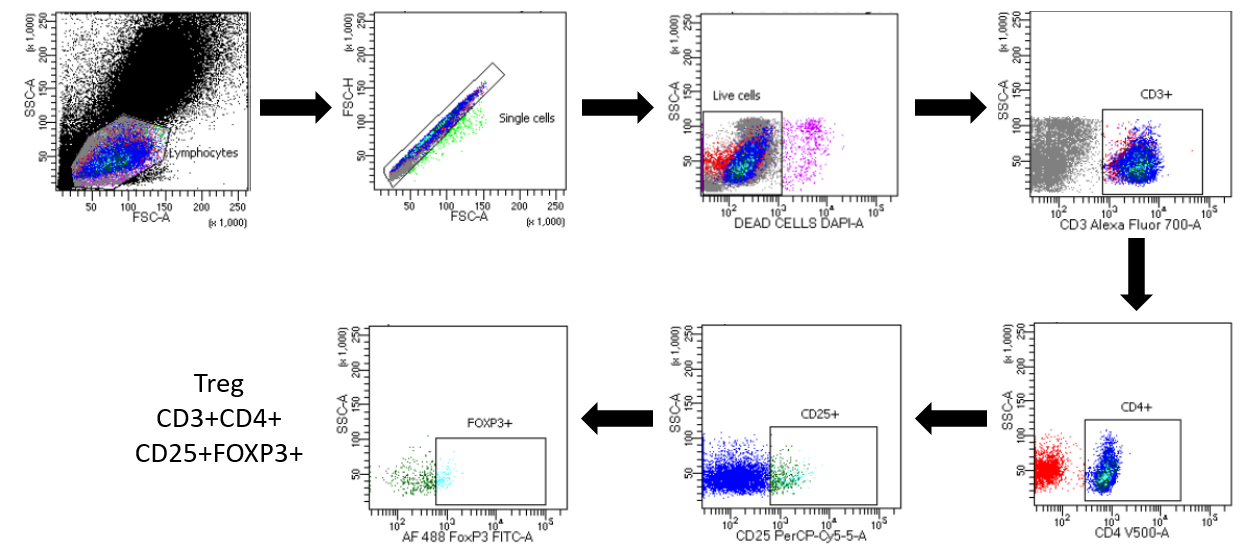
**

**Supplementary** **Figure 2**

Treg flow cytometry gating paradigm. Flow cytometry gating paradigm for the analysis of Tregs from control and PD patients. Peripheral blood is isolated from control and PD patients and stained for live cells (DAPI) and for lymphocyte markers CD3, CD4, CD25, and FOXP3. Tregs in our study are designated as live, single cells that express CD3+ CD4+ CD25+ FOXP3+ markers. Treg percentage is recorded as percent positive markers of total CD4+ cells.

**
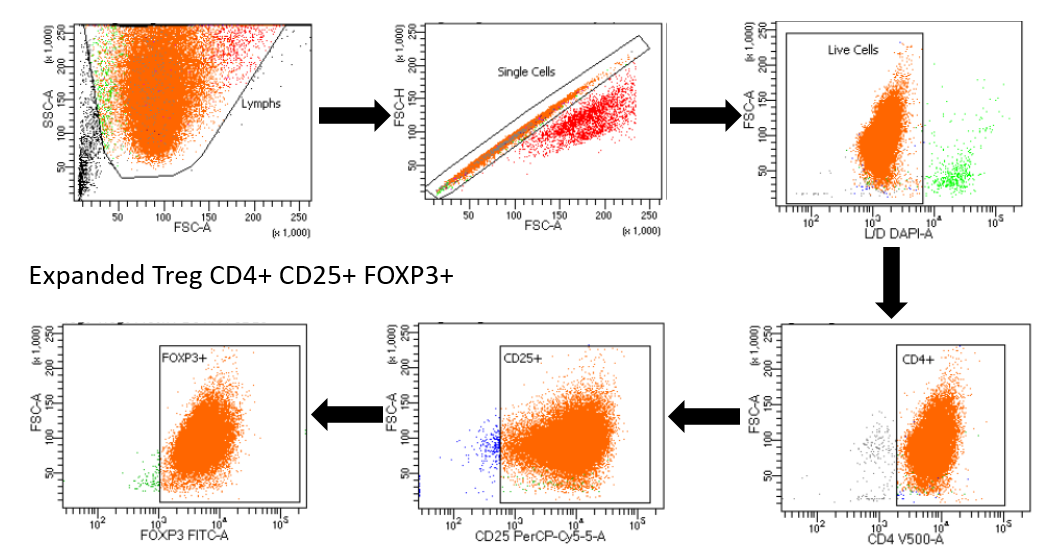
**

**Supplementary** **Figure 3**

Post-expanded Treg flow paradigm. Flow cytometric analysis of post-expanded Treg cells from patients and controls show an enrichment of live, single cells that are enriched in CD4+CD25+FOXP3+ signature.


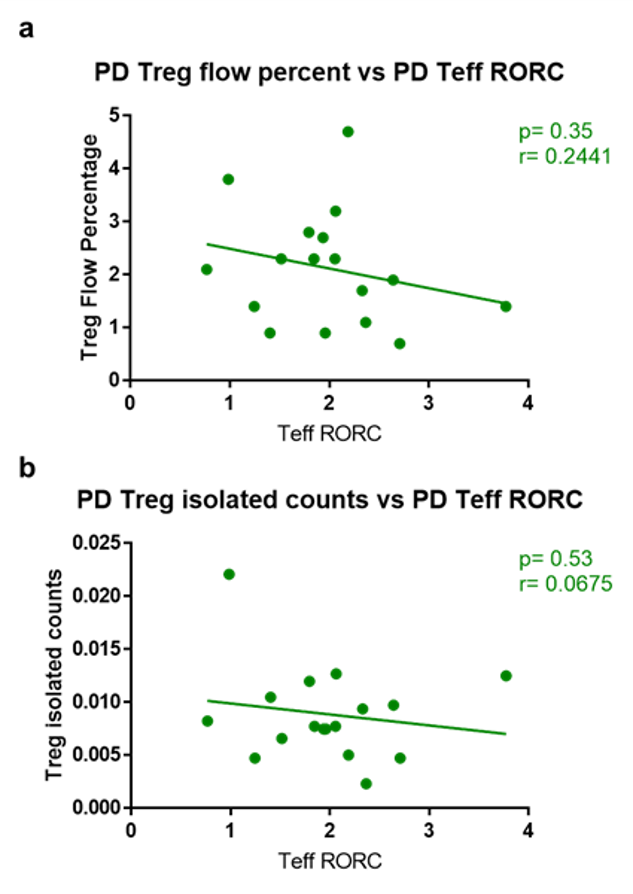


**Supplementary** **Figure 4**

Treg numbers and *rorc* transcription factor expression correlations. A reduction in PD Treg numbers analyzed via (a) flow and (b) magnetic isolation do not correlate with corresponding Teff *rorc* gene expression, the precursor gene for the Th17-polarizing transcription factor (PD patients, n=17).


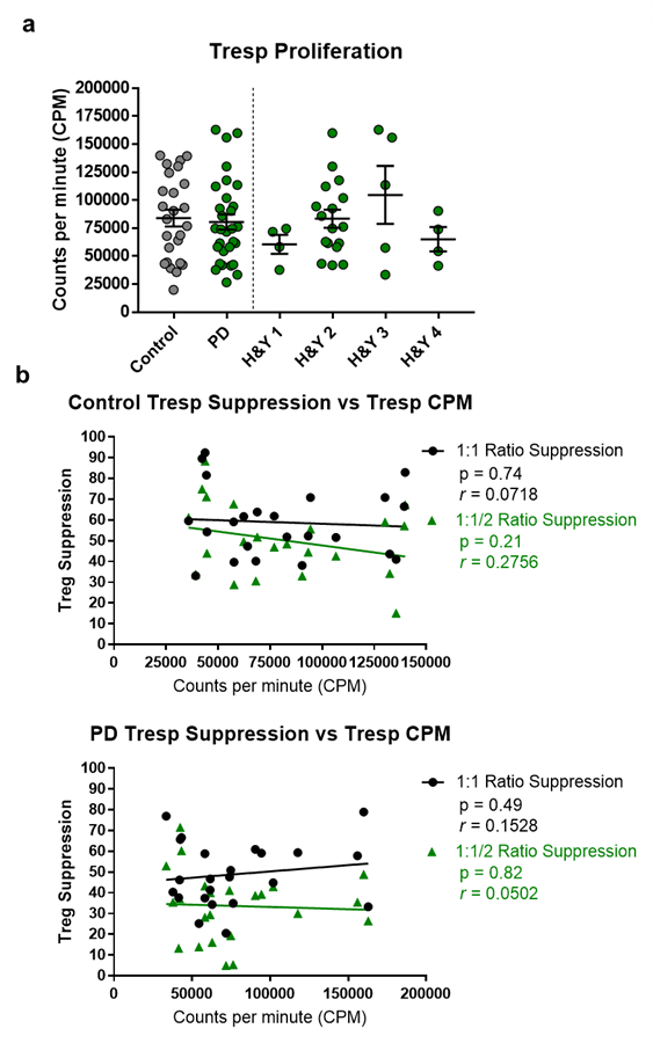


**Supplementary** **Figure 5**

T cell proliferation capacity in PD and control patients. The proliferative capacity in PD and controls is heterogenous with no difference between PD and controls or through PD progression measure by H&Y (C n= 25, PD n= 30). No correlation between Treg suppression and Tresp proliferative capacity.


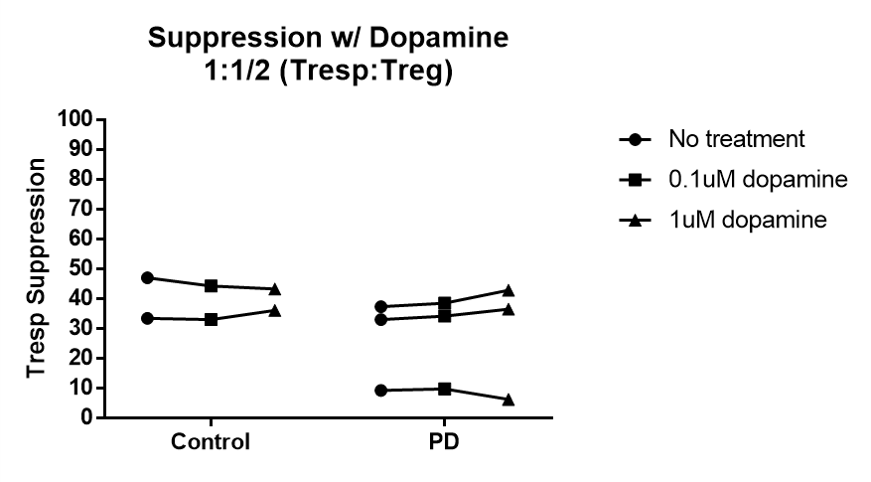


**Supplementary** **Figure 6**

No apparent effect of dopamine addition on Treg suppression. Dopamine addition to in vitro cultures did not affect Treg suppression in both control and PD co-culture systems of 1:1/2 Tresp:Treg proliferation assays.
