# Supplementary material for: Ex vivo expansion of dysfunctional regulatory T lymphocytes restores suppressive function in Parkinson’s disease
Source: NPJ Parkinsons Dis. 2021 May 13;7:41. doi: 10.1038/s41531-021-00188-5 (PMC8119976; doi:10.1038/s41531-021-00188-5)
Supplement: Supplementary file 2 — Reporting Summary [file 41531_2021_188_MOESM2_ESM.pdf]

nature research

Corresponding author(s): Stanley H. Appel NPJPARKD-00591R

Last updated by author(s): Jan 26, 2021

Reporting Summary

Nature Research wishes to improve the reproducibility of the work that we publish. This form provides structure for consistency and transparency in reporting. For further information on Nature Research policies, see our Editorial Policies and the Editorial Policy Checklist.

Statistics

For all statistical analyses, confirm that the following items are present in the figure legend, table legend, main text, or Methods section.

n/a	Confirmed	
☐	☒	The exact sample size (n) for each experimental group/condition, given as a discrete number and unit of measurement
☐	☒	A statement on whether measurements were taken from distinct samples or whether the same sample was measured repeatedly
☐	☒	The statistical test(s) used AND whether they are one- or two-sided<br>*Only common tests should be described solely by name; describe more complex techniques in the Methods section.*
☐	☒	A description of all covariates tested
☐	☒	A description of any assumptions or corrections, such as tests of normality and adjustment for multiple comparisons
☐	☒	A full description of the statistical parameters including central tendency (e.g. means) or other basic estimates (e.g. regression coefficient) AND variation (e.g. standard deviation) or associated estimates of uncertainty (e.g. confidence intervals)
☐	☒	For null hypothesis testing, the test statistic (e.g. F, t, r) with confidence intervals, effect sizes, degrees of freedom and P value noted<br>*Give P values as exact values whenever suitable.*
☒	☐	For Bayesian analysis, information on the choice of priors and Markov chain Monte Carlo settings
☒	☐	For hierarchical and complex designs, identification of the appropriate level for tests and full reporting of outcomes
☐	☒	Estimates of effect sizes (e.g. Cohen's d, Pearson's r), indicating how they were calculated

Our web collection on statistics for biologists contains articles on many of the points above.

Software and code

Policy information about availability of computer code

Data collection	No software was used.
Data analysis	No software was used.

For manuscripts utilizing custom algorithms or software that are central to the research but not yet described in published literature, software must be made available to editors and reviewers. We strongly encourage code deposition in a community repository (e.g. GitHub). See the Nature Research guidelines for submitting code & software for further information.

Data

Policy information about availability of data

All manuscripts must include a data availability statement. This statement should provide the following information, where applicable:

- Accession codes, unique identifiers, or web links for publicly available datasets
- A list of figures that have associated raw data
- A description of any restrictions on data availability

The data that supports the findings of this study are available from the corresponding author upon reasonable request.

Field-specific reporting

Life sciences study design

All studies must disclose on these points even when the disclosure is negative.

Sample size	The sample size utilized in our study for both the control and PD population are in accordance with numbers used in previously published reports in the field and reflect an acceptable sample size size for a cross-sectional analysis between PD and age-matched controls.
Data exclusions	No data were excluded from the manuscript unless an error occurred in sample processing which created uninterpretable results that could not be used in the analysis (example: flow cytometry acquisition issues). These events were rare and negligible for our study.
Replication	All data from control and PD populations were replicated in each experiment. We had did not come across issues of reproducibility and are confident with the outcomes of our findings.
Randomization	Patients from the PD population were recruited randomly with inclusion criteria of confirmed disease according to the Movement Disorder Society clinical diagnostic criteria for PD, age appropriate for disease, and exclusion criteria of no confounding immunological issues. Controls were recruited as age-matched with no confounding immunological issues.
Blinding	Investigators were aware of control or disease status during analysis of samples. Patient groupings were blinded during acquisition and experiments following Treg expansion were run using blinded, third party lab members.

Reporting for specific materials, systems and methods

We require information from authors about some types of materials, experimental systems and methods used in many studies. Here, indicate whether each material, system or method listed is relevant to your study. If you are not sure if a list item applies to your research, read the appropriate section before selecting a response.

Materials & experimental systems

n/a	Involved in the study	
☐	☒	Antibodies
☐	☒	Eukaryotic cell lines
☒	☐	Palaeontology and archaeology
☒	☐	Animals and other organisms
☐	☒	Human research participants
☒	☐	Clinical data
☒	☐	Dual use research of concern

Methods

n/a	Involved in the study	
☒	☐	ChIP-seq
☐	☒	Flow cytometry
☒	☐	MRI-based neuroimaging

Antibodies

Antibodies used	Flow cytometry antibodies: CD14 (eBioscience: 48-0149-42), CD16 (eBioscience: 11-0168-42), HLA-DR (eBioscience: 45-9956-42), CD33 (eBioscience: 17-0338-42), CD11b (eBioscience: 12-0118-42), CD3 (eBioscience: 56-0037-42), CD4 (BD: 560768), CD25 (BD: 560503), FOXP3 (eBioscience: 53-4776-42), CD8 (eBioscience 48-0088-42)
Validation	CD14 (eBioscience: 48-0149-42) https://www.thermofisher.com/antibody/product/CD14-Antibody-clone-61D3-Monoclonal/48-0149-42<br>CD16 (eBioscience: 11-0168-42) https://www.thermofisher.com/antibody/product/CD16-Antibody-clone-eBioCB16-CB16-Monoclonal/11-0168-42<br>HLA-DR (eBioscience: 45-9956-42) https://www.thermofisher.com/antibody/product/HLA-DR-Antibody-clone-LN3-Monoclonal/45-9956-42<br>CD33 (eBioscience: 17-0338-42) https://www.fishersci.com/shop/products/cd33-mouse-anti-human-apc-clone-wm-53-ebioscience/5014947<br>CD11b (eBioscience: 12-0118-42) https://www.thermofisher.com/antibody/product/CD11b-Antibody-clone-ICRF44-Monoclonal/12-0118-42<br>CD3 (eBioscience: 56-0037-42) https://www.fishersci.com/shop/products/anti-h-cd3-af700-100-t/5016845<br>CD4 (BD: 560768) https://www.bdbiosciences.com/us/applications/research/t-cell-immunology/th-1-cells/surface-markers/human/v500-mouse-anti-human-cd4-rpa-t4/p/560768<br>CD25 (BD: 560503) https://www.bdbiosciences.com/us/applications/research/b-cell-research/surface-markers/human/percp-cy55-mouse-anti-human-cd25-m-a251/p/560503<br>FOXP3 (eBioscience: 53-4776-42) https://www.thermofisher.com/antibody/product/FOXP3-Antibody-clone-PCH101-Monoclonal/53-4776-42<br>CD8 (eBioscience 48-0088-42) https://www.thermofisher.com/antibody/product/CD8a-Antibody-clone-RPA-T8-Monoclonal/48-0088-42<br>All flow antibodies were validated in our lab and appropriate IgG isotype was used.

Eukaryotic cell lines

Policy information about cell lines

Cell line source(s)	iPSC control line for generation of pro-inflammatory myeloid cells was obtained from the Induced Pluripotent Stem Cell Core at Cedars Sinai under the direction of Clive Svendsen, Ph.D. The iPSC line originated from the fibroblast of a 21 year old, clinically normal female patient.
Authentication	The IPSC line utilized in this study was provided by the Cedars Sinai iPSC core. Authentication measures can be obtained at the request of Cedars Sinai or referring to the bio-manufacturing page for this specific line (CS83iCTR): https://biomanufacturing.cedars-sinai.org/product/cs83ictr-33nxx/ .
Mycoplasma contamination	No mycoplasm contamination detected. More characterization of the cell line can be found here: https://biomanufacturing.cedars-sinai.org/product/cs83ictr-33nxx/
Commonly misidentified lines (See ICLAC register)	No misidentified cell lines were used in the study.

Human research participants

Policy information about studies involving human research participants

Population characteristics	PD patients (n=39, M/F: 27/12, age: 70.6±8.4) and age-matched healthy controls (n=31, M/F: 11/20, age: 69.5±8.9) were recruited to the study by the Houston Methodist Neurological Institute under the direction and evaluation of Dr. Eugene C. Lai and his Neurodegenerative Disease Clinic.
Recruitment	Patients and controls in our study were recruited by the Houston Methodist Neurological Institute under the direction and evaluation of Dr. Eugene C. Lai and his Neurodegenerative Disease Clinic. Patients for the study were selected as being PD positive according to the Movement Disorder Society clinical diagnostic criteria for PD, late-onset disease age appropriate, and devoid of confounding immunological issues. Controls were age-matched with no confounding immunological issues. Motor phenotypes of PD patients were evaluated using the Hoehn and Yahr (H&Y) scale which describes the motor manifestations of the disease according to stages (H&Y 1 to H&Y 5).
Ethics oversight	Written informed consent was obtained from PD patients and controls according to protocols evaluated and approved by the Houston Methodist Institutional Review Board (IRB).

Note that full information on the approval of the study protocol must also be provided in the manuscript.

Flow Cytometry

Plots

Confirm that:

- [x] The axis labels state the marker and fluorochrome used (e.g. CD4-FITC).
- [x] The axis scales are clearly visible. Include numbers along axes only for bottom left plot of group (a 'group' is an analysis of identical markers).
- [x] All plots are contour plots with outliers or pseudocolor plots.
- [x] A numerical value for number of cells or percentage (with statistics) is provided.

Methodology

Sample preparation	Immune cell populations were analyzed from peripheral blood and in vitro paradigms with a BD Bioscience LSR II bench top flow cytometer using immune cell fluorescent probes. Probes used for myeloid analysis include eBioscience anti-human: CD14-V450, CD16-FITC, HLA-DR-PerCP-Cy5.5, CD33-APC, and CD11b-PE. Monocyte populations are classified as follows: Classical monocytes (HLADR+CD14+CD16-), Intermediate monocytes (HLADR+CD14+CD16+), Non-classical monocytes (HLADR+CD14lowCD16+), and myeloid-derived suppressor cells (HLADR-CD14+CD11b+CD33+) (Supplemental Fig. 1). Identification of MDSCs based on suggested identification standards and previous lab studies investigating MDSCs.51,96,97 Lymphocyte population utilized anti-human: CD3-AF700 (eBioscience), CD4-v500 (BD Biosciences), CD25-PerCP-Cy5.5 (BD Biosciences), FOXP3-FITC (eBioscience), and CD8-v450 (eBioscience). Treg cells are classified as CD3+CD4+CD25+FOXP3+ cells (Supplemental Fig. 2). Viable cells were stained using Live/Dead Fixable Blue Dead Cell Stain Kit (Life Technologies) and appropriate isotype control antibodies were utilized according their respective fluorophore and company. For intracellular staining, cells were fixed and permeabilized using FoxP3/Transcription Factor Staining Buffer Set (eBioscience).
Instrument	BD LSRII Flow Cytometer configured with 355, 488, 405, 561, and 633 nm lasers
Software	BD FACSDiva software and DeNovo's FCS Express software.
Cell population abundance	Cell abundances of in our flow cytometry anaylsis corresponded with relative population frequencies expected from a peripheral blood analysis, particularly as it pertains to monocytes, myeloid-derived suppressor cells, and T cell populations such as CD3+, CD4+, CD8+ and Tregs (CD4+CD25+FOXP3+). No flow sorting of immune cell populations were done in this

study.

Gating strategy

Gating strategies are outlined in the paper and gating paradigms given in supplemental.
Briefly: Monocyte populations are classified as follows: Classical monocytes (HLADR+CD14+CD16-), Intermediate monocytes (HLADR+CD14+CD16+), Non-classical monocytes (HLADR+CD14lowCD16+), and myeloid-derived suppressor cells (HLADR-CD14+CD11b+CD33+). Identification of MDSCs based on suggested identification standards and previous lab studies investigating MDSCs. T cell populations are determined as being live/single cells that are CD3+ and CD8+ for cytotoxic T cells, CD4+ for T helper cells, CD4+CD25- for T effector cells, and Treg cells are classified as CD3+CD4+CD25+FOXP3+ cells.

☒ Tick this box to confirm that a figure exemplifying the gating strategy is provided in the Supplementary Information.
